# Multiscale Dynamics of Blood Pressure Fluctuation Is Associated With White Matter Lesion Burden in Older Adults With and Without Hypertension: Observations From a Pilot Study

**DOI:** 10.3389/fcvm.2021.636702

**Published:** 2021-02-26

**Authors:** Xin Jiang, Yi Guo, Yue Zhao, Xia Gao, Dan Peng, Hui Zhang, Wuhong Deng, Wen Fu, Na Qin, Ruizhen Chang, Brad Manor, Lewis A. Lipsitz, Junhong Zhou

**Affiliations:** ^1^Department of Geriatrics, Shenzhen People's Hospital, Shenzhen, China; ^2^The Second Clinical Medical College, Jinan University, Shenzhen, China; ^3^The First Affiliated Hospital, Southern University of Science and Technology, Shenzhen, China; ^4^Department of Neurology, Shenzhen People's Hospital, Shenzhen, China; ^5^Shenzhen Bay Laboratory, Shenzhen, China; ^6^Department of Electrical and Computer Engineering, University of Rochester, Rochester, NY, United States; ^7^Hinda and Arthur Marcus Institute for Aging Research, Hebrew SeniorLife, Boston, MA, United States; ^8^Division of Gerontology, Beth Israel Deaconess Medical Center and Harvard Medical School, Boston, MA, United States

**Keywords:** hypertension, beat-to-beat blood pressure fluctuation, multiscale entropy, white matter lesions, older adults

## Abstract

**Background:** White matter lesions (WMLs) are highly prevalent in older adults, and hypertension is one of the main contributors to WMLs. The blood pressure (BP) is regulated by complex underlying mechanisms over multiple time scales, thus the continuous beat-to-beat BP fluctuation is complex. The association between WMLs and hypertension may be manifested as diminished complexity of BP fluctuations. The aim of this pilot study is to explore the relationships between hypertension, BP complexity, and WMLs in older adults.

**Method:** Fifty-three older adults with clinically diagnosed hypertension and 47 age-matched older adults without hypertension completed one MRI scan and one BP recording of 10–15 min when sitting quietly. Their cerebral WMLs were assessed by two neurologists using the Fazekas scale based on brain structural MRI of each of their own. Greater score reflected higher WML grade. The complexity of continuous systolic (SBP) and diastolic (DBP) BP series was quantified using multiscale entropy (MSE). Lower MSE reflected lower complexity.

**Results:** Compared to the non-hypertensive group, hypertensives had significantly greater Fazekas scores (*F* > 5.3, *p* < 0.02) and lower SBP and DBP complexity (*F* > 8.6, *p* < 0.004). Both within each group (β < −0.42, *p* < 0.01) and across groups (β < −0.47, *p* < 0.003), those with lower BP complexity had higher Fazekas score. Moreover, complexity of both SBP and DBP mediated the influence of hypertension on WMLs (indirect effects > 0.25, 95% confidence intervals = 0.06 – 0.50).

**Conclusion:** These results suggest that diminished BP complexity is associated with WMLs and may mediate the influence of hypertension on WMLs. Future longitudinal studies are needed to examine the causal relationship between BP complexity and WMLs.

## Introduction

White matter lesions (WMLs), as characterized by a hyperintense signal on T2-weighted or fluid-attenuated inversion recovery (FLAIR) MRIs (1), are highly prevalent in older adults (2). WMLs are considered a sign of cerebral microvascular diseases and have been linked to dementia (3), depression (4), and slowed gait (5) in older adults. Cardiovascular abnormalities, including hypertension, play a key role in the formation of WMLs by possibly damaging the cerebral microcirculation (6). Previous studies showed that, for example, hypertension is strongly associated with WML, that is, compared to age-matched non-hypertensive individuals, older adults with hypertension had a greater burden of WMLs (7, 8). However, the underlying mechanism through which hypertension contributes to WMLs is not fully understood.

Multiple underlying physiologic components, consisting of numerous neural feedback and circadian rhythmic changes, are involved in the regulation of blood pressure (BP) (9, 10). These components are communicating and interacting with each other non-linearly over multiple temporal–spatial scales. As such, the multiscale dynamics of the continuous beat-to-beat BP fluctuation are “complex,” meaning that the BP fluctuation contains information-rich and fractal-like patterns that are self-similar over multiple scales of time (9). The degree of such physiologic complexity can be captured using metrics derived from the theory of complex systems, such as multiscale entropy (MSE). The MSE is one well-developed and widely used technique that quantifies the recurrence of patterns, or entropy, in physiologic series at different scales of time or space (11). Studies have shown that the greater the likelihood of recurrent patterns (e.g., as occurs in a sine wave), the less complex the fluctuation, the poorer the system's adaptability and functionality. Zhou et al. (12), for example, have recently shown that lower complexity of resting-state brain activity (i.e., blood oxygen level-dependent signal of functional MRI), as quantified using MSE, is associated with slower walking speed and less integration of brain white matter in older adults. Still, the influences of hypertension on complexity of the beat-to-beat BP fluctuation remain unclear, and the contribution of BP complexity to WMLs is unknown. We here contend that the association between hypertension and WMLs would be manifested as a loss of complexity of the continuous BP fluctuations.

Specifically, we hypothesize in the current study that (1) compared to those without hypertension (i.e., non-hypertensive group), older adults with hypertension (i.e., hypertensive group) would have lower complexity in both beat-to-beat systolic and diastolic BP (SBP and DBP) fluctuation; (2) such lower complexity would be associated with a greater grade of WMLs in both hypertensive and non-hypertensive older adults; and (3) the BP complexity would mediate the influence of hypertension on WMLs.

## Methods

### Participants

We completed information search in a data repository (from year 2019 to 2020) for older adults (i.e., age >60 years) who had a clinical visit within the previous 12 months in the Department of Geriatrics, Shenzhen People's Hospital, and expressed interest in participating future studies. A total of 170 records, including 100 *with clinically diagnosed hypertension* (i.e., SBP ≥140 mmHg and/or DBP ≥80 mmHg by measuring the brachial artery of right arm using sphygmomanometer) and 70 *without hypertension*, were found without any selection bias. Two clinicians and one study personnel then contacted them and completed the screening on those who can participate in this study. The exclusion criteria for both cohorts included terminal disease, diagnosis of dementia or other overt neurological diseases (e.g., Parkinson's disease or stroke), obesity (i.e., BMI ≥30 kg/m^2^), chronic kidney disease, dyslipidemia, other cardiovascular diseases (e.g., heart failure, coronary artery disease), any condition making it difficult to perform the MRI (e.g., metal implant in the brain), physical dysfunction (i.e., cannot stand or walk for 30 s without personal assistance), history of brain trauma or injury, use of medications that may alter the cardiovascular and cerebrovascular function (except for antihypertension medications that were used in the hypertensive group to control BP), and inability to understand the study protocol. All experimental methods and protocols were approved by the institutional review board (IRB) of Shenzhen People's Hospital and carried out in accordance with relevant guidelines. All the participants provided written consent in order to participate in the study.

After the screening, a total of 100 eligible older adults (i.e., 53 hypertensives and 47 non-hypertensives) completed the MRI scans and BP recordings. Hypertension had been controlled using antihypertension medications, and the number of antihypertensive medications they used and the left brachial-ankle pulse wave velocity (baPWV), a metric to assess the degree of arterial stiffness, were assessed and measured during their clinical visit and included in the following analyses (13).

### Study Protocol

#### MRI and Fazekas Scale

Each participant completed the structural MRI scan consisting of T1, T2, and FLAIR sequences using a 1.5-Tesla MR scanner. The specific parameters for the MRI scan were: 3D-FLAIR: repetition time (TR)/echo time (TE)/inversion time (TI) = 9,000/2,500/85 ms, slice thickness = 5 mm, echo train length (ETL) = 16, matrix size = 512 × 512 mm; T1: TR/TE = 250/2.48 ms, slice thickness = 5 mm, ETL = 1, matrix size = 320 × 320 mm; and T2: TR/TE = 6,000/99 ms, slice thickness = 5 mm, ETL = 18, matrix size = 640 mm × 640 mm.

The grade of WMLs was then assessed separately by two neurologists using the Fazekas scale based upon the brain structural MRI of each participant (14). Both neurologists were blinded to the hypertensive status and other demographic and clinical information of each participant. The Fazekas scale divided the white matter into periventricular and deep white matter, and each region was scaled at a grade ranging from 0 to 3 (i.e., four grades) depending on the size and confluence of lesions. The grade of WMLs was scored primarily on the FLAIR images, and the T2 and T1 images were also used when necessary. If there was a difference between the scales given by the two neurologists, the scale was determined and approved after a careful discussion between them (only five cases were assessed differently). The scale of the whole white matter (i.e., the sum of WML grades in periventricular and deep white matter) ranged from 0 to 6 and was used in the following analysis. Greater Fazekas scale reflected higher grade of WMLs.

#### Blood Pressure Recordings

The BP assessment was completed in a quiet room, where only the participant and one study administrator were present. Each participant completed a BP assessment when sitting quietly after at least a 30-min break following the MRI. During the assessment, the participant was prevented from talking and was instructed to be motionless (e.g., not crossing arms). Other devices that may interrupt the assessments, such as cellphones, were also stored in a secured box outside the assessment room. The continuous finger BP, including the SBP, and DBP fluctuation, was measured non-invasively using Finometer PRO system (Finapres Medical Systems B.V., Netherlands) at the middle finger of the left hand in supine position. The beat-to-beat BP and inter-beat interval were recorded continuously for at least 10 min (10–15 min) and sampled at 100 Hz. The BeatScope software package (Finapres Medical Systems B.V., Netherlands) calculated the values of SBP and DBP of each beat. This type of finger BP measurement was validated against invasive brachial artery pressure (15). All the recordings thus consisted of at least 700 continuous BP beat samples. The outliers in the BP series of which the value was greater or lower than mean ± 2 × standard deviation (SD) of the series were interpolated by the mean. The preprocessed BP series with the length of 700 points was then used in the following analyses.

### Multiscale Entropy

**Figure 1:**
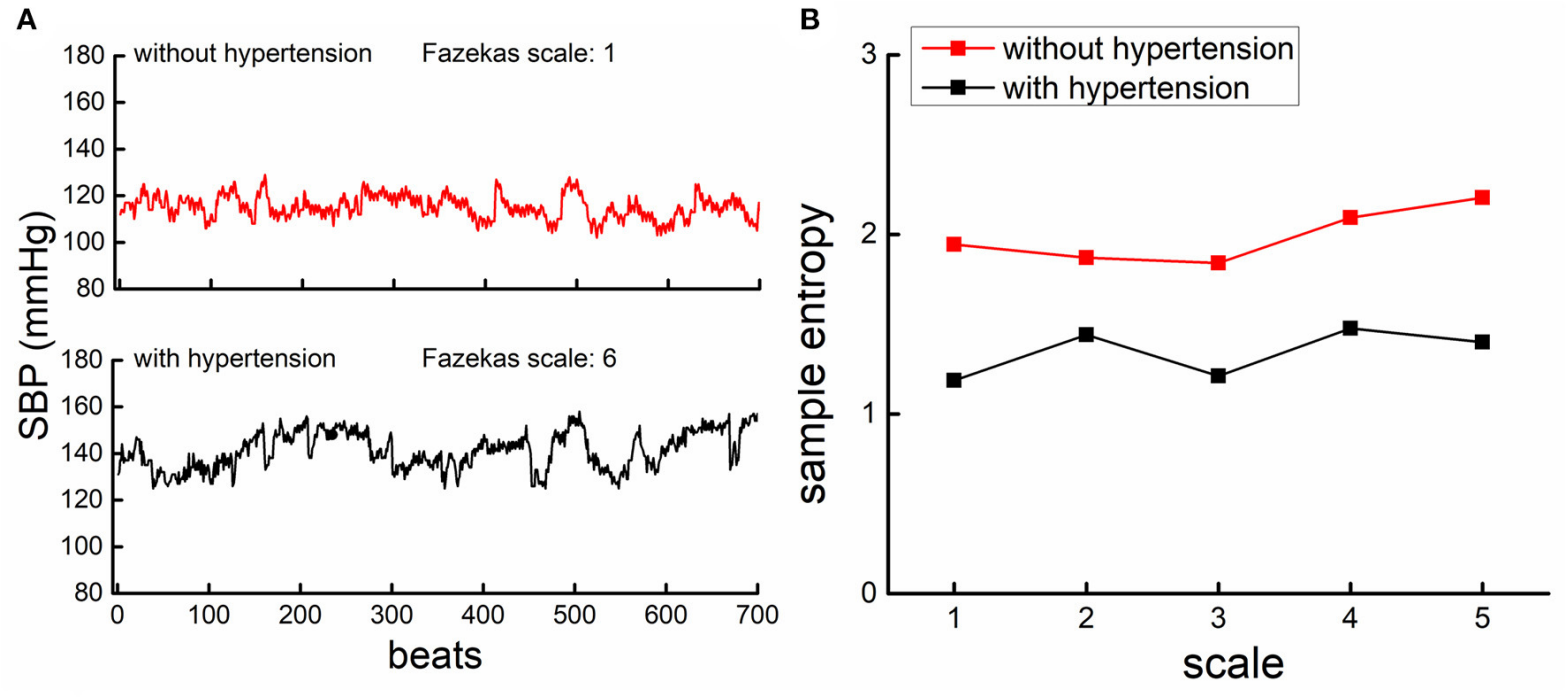
The beat-to-beat systolic blood pressure (SBP) series from a participant with hypertension [lower panel of **(A)**, black] had lower complexity than the participant without hypertension [upper panel of **(A)**, red]. **(A)** An example of the beat-to-beat SBP series from one participant without hypertension (upper left panel) and from one participant with hypertension (lower left panel). **(B)** Shows the results of multiscale entropy (MSE) from the SBP series of these two participants. The entropies of the participant with hypertension were lower than those of the participant without, across all the five scales, indicating that the SBP fluctuation was less complex in hypertensive as compared to non-hypertensive.

The complexity of SBP and DBP series was quantified using MSE, a well-established measure of the non-linear dynamics in the fluctuation of physiological series. The MSE divided the time-series into non-overlapping windows of length equaling to a scale factor, τ, and then calculated the sample entropies of each “coarse-grained” series. Here, we used scales ranging from 1 to 5 data points. Thus, in the coarse-graining process, the series at Scale 1 was the raw time-series consisting of 700 data points, that at Scale 2 was constructed by averaging every two non-overlapped points, consisting of 350 points (i.e., 700 points/2), and that at the largest scale (i.e., Scale 5) had 140 data points (i.e., 700 points/5), meeting the standard practice for obtaining reliable estimates of sample entropy (16). Sample entropy was defined by the negative natural logarithm of the conditional probability that a time-series, having repeated itself within a tolerance r for m points (pattern length), also repeated itself for m + 1 points without self-matches. The sample entropy of each coarse-grained series in this study was computed by choosing *m* = 2 and *r* = 15%, which was suggested by previous studies (16, 17). Figure 1B showed the example of MSE curves of BP series generated by plotting the sample entropy as a function of time scales from the series of two participants as presented in Figure 1A (upper panel: one without hypertension and with Fazekas scale of 2; lower panel: one with hypertension and with Fazekas scale of 6). Finally, the BP complexity was defined as the averaged entropy across five scales. Lower averaged entropy reflected lower complexity. To ensure the validity of this MSE-based measure, we performed the validation tests of the complexity metric as shown in Supplementary Materials.

Additionally, the mean and variability of SBP, DBP, and pulse pressure (PP) were also obtained by calculating the mean and the coefficient of variability (i.e., the ratio of SD to mean) of continuous BP fluctuations, respectively, and used in the following analyses.

### Statistical Analysis

Statistical analyses were performed with JMP Pro 14 software (SAS Institute, Cary, NC) and SPSS 20 (IBM Corp., Armonk, NY). The significance level was set at *p* < 0.05. The Shapiro–Wilk test was used to test if the outcomes were normally distributed.

To examine the effects of hypertension on BP complexity, two-way ANOVA models were used. The dependent variables of each model were the complexity of SBP and DBP, respectively, and the factor was group (i.e., hypertensive and non-hypertensive). The effects of hypertension on the Fazekas scale and mean and variability of SBP, DBP, and PP were also examined using similar models. Age, sex, BMI, cognitive function, education, smoking status, duration of hypertension, type of antihypertensive medication, number of antihypertensive medications, baPWV, and the presence of diabetes (i.e., if they had diabetes or not) were used as covariates in these ANOVA models, and their effects were also assessed.

To explore the relationship between BP complexity and WMLs, the total score of Fazekas scale was used. We used linear regression models to test if the complexity of SBP and DBP was correlated with the Fazekas scale across the entire cohort and within each of the hypertensive and non-hypertensive cohort. Age, sex, BMI, cognitive function, education, smoking status, duration of hypertension, type of antihypertensive medication, number of antihypertensive medications, mean and variability of BP (i.e., SBP, DBP, and PP), baPWV, and the presence of diabetes were used as covariates in these regression models, as all of them may potentially contribute to the degree of BP complexity and the grade of WMLs. Similar models were used to examine the relationships between the Fazekas scale and BP level and variability, between the BP complexity and mean and variability of BP, and between the SBP complexity and DBP complexity by using age, sex, BMI, cognitive function, education, smoking status, duration of hypertension, type of antihypertensive medication, number of antihypertensive medications, baPWV, and the presence of diabetes as covariates.

To test the hypothesis that the BP complexity would mediate the relationship between hypertension and WMLs, we utilized *mediation analyses*. The complexity of SBP and DBP was used as mediators in separate models. The dependent variable was the Fazekas scale of the whole white matter, and the independent variable was the category of BP status (i.e., “hypertensive” or “non-hypertensive”). We calculated the total effects of the BP status on WMLs (i.e., total effect, path c) and the association between BP status and the BP complexity (path a). Then, we examined the association between BP complexity and WMLs (path b), which provided the estimates for the direct effects (path c'). The percentage of mediation (i.e., P_M_) was determined by dividing the indirect effect (path a × path b) by the total effect (path c). A bootstrapping method with *N* = 5,000 bootstrap samples was used to calculate the 95% Bias Corrected and Accelerated Confidence Interval (95% BCa CIs) around the mediated and direct effects. Age, sex, BMI, cognitive function, education, smoking status, duration of hypertension, mean and variability of BP (i.e., SBP, DBP, and PP), baPWV, the type of antihypertension medication, number of antihypertensive medications, and the presence of diabetes were used as covariates in the mediation analyses.

## Results

Table 1 showed the demographics and clinical characteristics of the entire cohort and of the two groups (i.e., hypertensive and non-hypertensive cohorts) separately. Within the hypertensive group, five types of antihypertension medication were used, that is, calcium channel blockers (CCB), angiotensin-converting enzyme inhibitors (ACEIs), angiotensin-II receptor blockers (ARBs), beta-blockers (BB), and diuretics. Among them, the number of antihypertension medications they were using at the same time was different. Specifically, 25 of the hypertensive participants used only one type of medication, 19 of them used two types, seven of them used three types, and two of them used four types of medication. No participants in the non-hypertensive group took antihypertension medication. The Shapiro–Wilk test showed that all the outcomes were normally distributed (*p* > 0.24). Hypertensive group had a higher baPWV as compared to that of the non-hypertensive group (*p* = 0.03). No significant difference in other demographic and clinical characteristics was observed between two groups.

**Table 1:** The demographics, white matter lesions, anti-hypertensive medication information and BP metrics of participants.

**Mean** **±** **S.D**.		**Total cohort** **(*n* = 100)**	**Hypertension cohort** **(*n* = 53)**	**Non-hypertension cohort** **(*n* = 47)**	***p***
Age (years)		74.4 ± 8.8	73.7 ± 9.2	70.4 ± 9.8	0.50
Sex (female)		*n* = 50	*n* = 27	*n* = 23	0.96
BMI		23.8 ± 3.2	23.7 ± 2.2	23.8 ± 2.4	0.22
Education (years)		11.7 ± 3.8	11.8 ± 2.7	11.5 ± 4.2	0.61
Presence of diabetes (*n*)		40	13	11	0.97
Smoker (*n*)		15	7	8	0.98
MMSE		27.4 ± 3.6	25.2 ± 4	27.8 ± 1.9	0.25
baPWV (cm/s)		1,829 ± 416	1,902 ± 474	1,810 ± 356	0.03
Duration of hypertension (years)		12 ± 8	12 ± 8	n.a.	n.a.
Anti-hypertensive medication (*n*)	CCB	30	30	n.a	n.a
	ACEI	7	6	n.a	n.a
	ARB	22	22	n.a	n.a
	BB	21	21	n.a	n.a
	Diuretics	9	9	n.a	n.a
Number of medications	1	25	25	n.a	n.a
	2	29	29	n.a	n.a
	3	7	7	n.a	n.a
	4	2	2	n.a	n.a
Fazekas scale	Total	2.5 ± 1.4	2.9 ± 1.3	1.9 ± 1.5	0.003
	Periventricular	1.3 ± 0.8	1.5 ± 0.7	1.1 ± 0.9	0.02
	Deep	1.2 ± 0.8	1.4 ± 0.8	0.9 ± 0.8	0.01
Mean level	SBP	135 ± 17.3	142.9 ± 14.9	124.5 ± 14.7	<0.0001
	DBP	77.5 ± 8.9	79.8 ± 9.4	74.3 ± 6.9	0.01
	PP	57.2 ± 8.8	60.5 ± 7.6	55.8 ± 6.3	<0.0001
Variability	SBP	0.04 ± 0.01	0.04 ± 0.01	0.04 ± 0.01	0.81
	DBP	0.05 ± 0.02	0.05 ± 0.03	0.04 ± 0.01	0.69
	PP	0.06 ± 0.02	0.05 ± 0.01	0.04 ± 0.02	0.43
Complexity	SBP	1.45 ± 0.28	1.38 ± 0.25	1.55 ± 0.28	0.004
	DBP	1.37 ± 0.32	1.26 ± 0.31	1.53 ± 0.27	0.0001

*BMI, body mass index; CCB, calcium channel blockers; ACEI, angiotensin-converting enzyme inhibitors; ARB, angiotensin II receptor blockers; BB, beta-blockers; baPWV, brachial-ankle pulse wave velocity; SBP, systolic blood pressure; DBP, diastolic blood pressure; PP, pulse pressure. n.a., not available*.

### The Effects of Hypertension on Blood Pressure Complexity and White Matter Lesions

Figure 1 showed the SBP series from one participant with hypertension (lower panel of **A**) and one participant without hypertension (upper panel of A), along with the MSE curves generated from each SBP series (**B**). As compared to the non-hypertensive group, the sample entropy of the SBP series in the hypertensive group was lower across multiple scales of time. As such, BP complexity (i.e., the averaged entropy across five scales) of this participant was considerably less than the non-hypertensive participant. At the group level, we observed that compared to the non-hypertensive group, the hypertensive group had significantly lower complexity of SBP (*F* = 8.3, *p* = 0.003) and DBP (*F* = 16.1, *p* = 0.0001) and, as expected, had a higher mean level of SBP (*F* = 18.9 *p* < 0.0001), DBP (*F* = 6.9, *p* = 0.01), and PP (*F* = 7.8, *p* < 0.003). But no significant difference was observed between the BP variability in the hypertensive and non-hypertension groups (*F* < 0.12, *p* > 0.43; Table 1). On the other hand, we observed that the grade of WMLs, as quantified by the Fazekas scale, was significantly higher in the hypertensive group as compared to the non-hypertensive group (*F* = 9.6, *p* = 0.003; Table 1). All the significance was independent of age, sex, BMI, education, cognitive function, smoking, duration of hypertension, baPWV, types of antihypertension medication, number of antihypertensive medications, and the presence of diabetes, and no significant effects of these covariates (e.g., the presence of diabetes: *F* < 0.08, *p* > 0.43) were observed on BP complexity and grade of WMLs (*F* < 0.1, *p* > 0.24).

### The Relationship Between Blood Pressure Complexity and White Matter Lesions

**Figure 2:**
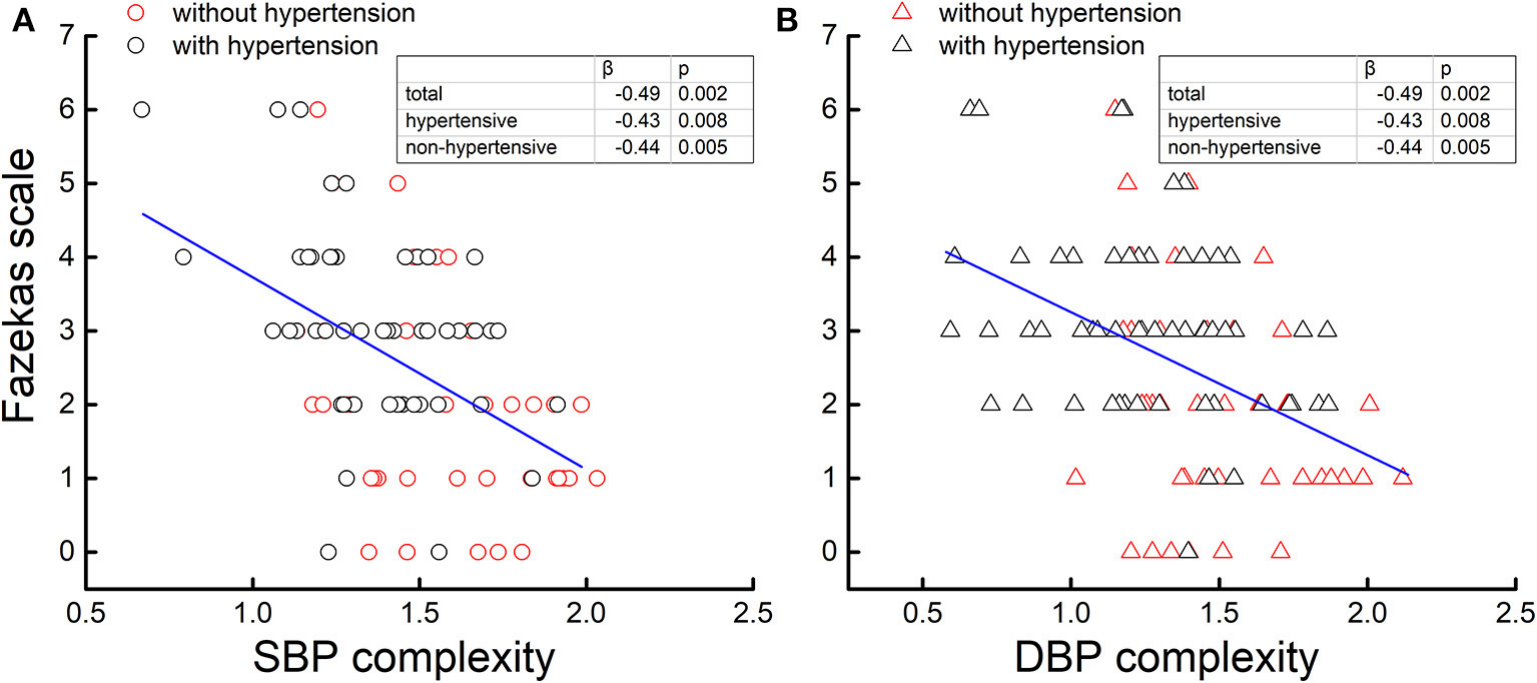
The complexity of the systolic blood pressure (SBP) **(A)** and diastolic blood pressure (DBP) **(B)** series was associated with the Fazekas scale. Across two cohorts [hypertensive (black) and non-hypertensive (red)], participants with lower BP complexity had a greater Fazekas scale (β = −0.49, *p* < 0.002), that is, a higher grade of cerebral white matter lesions (WMLs), while no association was observed between the BP level and variability and WMLs. Moreover, within each cohort (i.e., hypertensive and non-hypertensive), such associations were still present (β < −0.43, *p* < 0.008).

The linear regression analyses demonstrated that the complexity of SBP and DBP was not associated with the level or variability of SBP or DBP, respectively (β <0.15, *p* > 0.18), but those with lower SBP complexity also had lower DBP complexity (β > 0.65, *p* < 0.0001). Across the entire cohort, the SBP and DBP complexity was each associated with the Fazekas scale, that is, participants with lower SBP (β = −0.49, *p* = 0.002; Figure 2A) and/or lower DBP (β = −0.49, *p* = 0.002; Figure 2B) complexity had a greater Fazekas scale (i.e., higher WML grades). *Within each group* (i.e., hypertensive and non-hypertensive), such significant associations were also observed, that is, participants with lower SBP and/or lower DBP complexity had a greater Fazekas scale (β < −0.43, *p* < 0.008; Figure 2). All the significance was independent of age, sex, BMI, mean and variability of BP, education, cognitive function, smoking status, duration of hypertension, baPWV, the types of antihypertension medication, number of antihypertensive medications, and the presence of diabetes. On the other hand, no significant associations were observed between the mean and variability of SBP and DBP and the Fazekas scale (β <0.4, *p* > 0.12). Within the non-hypertensive group, the mean of PP was associated with the Fazekas scale (β = 0.3, *p* = 0.03), but within the hypertensive group, such association was not observed (β = 0.01, *p* = 0.69).

### Mediation Effects of Blood Pressure Complexity on the Relationship Between Hypertension and White Matter Lesions

**Figure 3:**
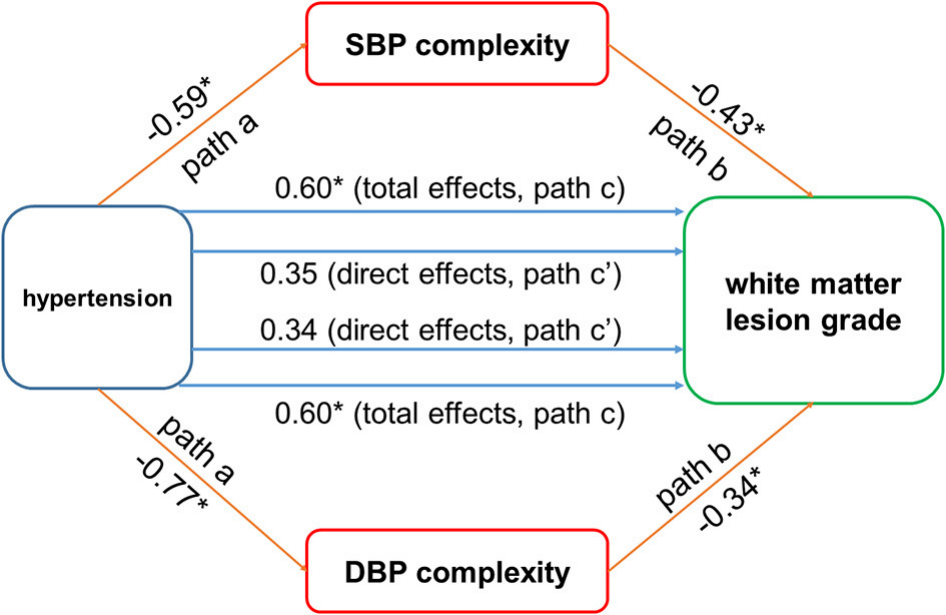
The blood pressure (BP) complexity mediated the relationship between hypertension and cerebral white matter lesions (WMLs). Hypertension was negatively associated with systolic blood pressure (SBP) and diastolic blood pressure (DBP) complexity (paths a; β < −0.59, *p* < 0.008). The BP complexity was associated with WMLs (path b: β < −0.34, *p* < 0.003). Significant total effect of hypertension on WMLs was observed (path c; β = 0.60, *p* = 0.006), while the direct effects (path c') of hypertension on WMLs were not significant after using the BP complexity as mediator (SBP complexity: β = 0.34, *p* = 0.10; DBP complexity: β = 0.49, *p* = 0.12). *p < 0.01.

The mediation procedures revealed that the influence of hypertension on the grade of WMLs was mediated by both SBP and DBP complexity (Figure 3). Specifically, the hypertension was associated with SBP and DBP complexity (path a; β < −0.59, *p* < 0.008). Though the total effects of hypertension on the grade of WMLs, as quantified by the Fazekas scale, were statistically significant (path c; β = 0.60, *p* = 0.006), the direct effects of hypertension on the grade of WMLs were no longer significant after introducing BP complexity as mediator (path c'; SBP complexity: β = 0.34, *p* = 0.10; DBP complexity: β = 0.35, *p* = 0.12). Separate mediation models showed that SBP complexity accounted for 43% of the total effects on the grade of WMLs [indirect effects (i.e., path a × path b) = 0.26, mediated proportion (P_M_) = 43%, 95% bias-corrected and accelerated confidence interval (Bca CI) = 0.06 – 0.50], and DBP complexity accounted for 43% of the total effects (indirect effects = 0.25, *P*_*M*_ = 43%, 95% *Bca CI* = 0.09 – 0.49).

## Discussion

Most previous research efforts put focus on the mean BP level and/or its variability over a long time (e.g., over days or years) (18, 19). The regulation of BP, however, is continuous and dynamic across multiple scales of time and space. This pilot study is the first to demonstrate that: (1) the complexity in the *multiscale dynamics* of continuous beat-to-beat BP fluctuation is diminished in hypertensive older adults; (2) the complexity of SBP and DBP was associated with the grade of WMLs; and (3) such BP complexity may mediate the relationship between hypertension and WMLs. These results indicate that the physiologic complexity of continuous beat-to-beat BP fluctuation can provide unique insights into the regulation of BP and may indicate a potential mechanism through which hypertensive status in the cardiovascular system affects the cerebral characteristics in older adults.

BP is determined by the cardiac output and the systemic vascular resistance and is maintained within a certain range by multiple underlying neural and hormonal feedback mechanisms, such as baroreflex mechanism (20). The baroreflex mechanism consists of a host of neurophysiologic components, including resistance vessels, baroreceptors, and sympathetic and parasympathetic nervous systems (21). These components are functioning and interacting with each other non-linearly with varied time delays, such that the dynamics of fluctuation in the beat-to-beat BP series are complex. In the “complexity theory of aging,” aging and age-related conditions often alter the quantity and/or quality of the regulatory elements of a physiologic system as well as their structural and functional connections within the system, which manifests as a loss of the physiologic complexity in the dynamics of the system's output (22). Previous studies have linked the age-related decrease of physiologic complexity in the cardiovascular system to cardiovascular diseases and mood disorders (23, 24). However, the multiscale dynamics of beat-to-beat BP fluctuation have not been well-characterized. More recently, a few studies measuring the complexity of beat-to-beat BP fluctuations have emerged and observed the association between BP complexity and disease and cardiac risk events (25, 26). Henriques et al. (25), for example, demonstrated that lower preoperative BP complexity, as quantified using MSE, was associated with a higher estimated risk of adverse cardiovascular outcomes during surgery. We here provide novel evidence that lower BP complexity may capture the loss of functionality in the cardiovascular system due to hypertension, which may also be pertaining to the impaired regulation of cerebral blood flow that contributes to WMLs in older adults.

In this study, we observed that BP complexity mediated the established association between hypertension and WML grade, contributing to over 43% of this association. Hypertension is one of the key contributors to WMLs in older adults, oftentimes leading to the thickening of vessel walls with narrowing of lumen and hyalinosis of the media. Such deterioration induced by hypertension then results in stiffness and tortuous elongation of the small cerebral vessels (27–29). These structural alterations impair cerebral autoregulation and elevate the hypoperfusion, ultimately increasing the grade of WMLs in older adults. Our mediation findings may reveal a complementary but novel conceptual framework: in addition to the impairments on cerebral vascular function, hypertension alters BP regulation by disrupting the functionality of the cardiovascular system (as captured by lower BP complexity), and such disruption diminishes the capacity of blood circulation system to regulate the blood supply to cerebral regions efficiently and effectively, leading to an elevated grade of WMLs in older adults.

We also observed no association between mean or variability of BP and WMLs except that between mean PP and WMLs within the non-hypertensive group. These traditional metrics reflect the regulatory behavior of BP on only a single scale of time, which may thus not fully capture the system functionality of BP regulation. The BP complexity, however, was significantly associated with the grade of WMLs, and such association was independent of multiple factors, including the use of antihypertension medication, duration of hypertension, etc. Additionally, the BP complexity in older adults without hypertension is associated with the WML grade. Taken together, it may indicate that as compared to traditional BP measures (e.g., BP level), the BP complexity quantifying the multiscale dynamics of BP fluctuation may be a marker that is sensitive to subtle changes in the vascular system even when no significant conditions or diseases (e.g., hypertension) are presented in older adults. However, it should be noted that the findings of this study are based upon cross-sectional analyses; it is thus warranted to explore the longitudinal relationship between changes in BP complexity and the progress of WMLs in older adults, as well as the incidence of other diseases pertaining to cerebral dysfunction, such as dementia.

Though exciting, the current study has limitations. The sample size (*n* = 100) in this pilot study was still relatively small, and only a single measure of BP fluctuation was used. Future work with a larger sample size using repeated measurement of BP fluctuation is thus needed to confirm the findings in this study and test the reproducibility of the measurement. One important limitation is that the participants were recruited from a data repository and the BP in the hypertensive group had been controlled using antihypertension medications for a certain time. Though the use of antihypertension medication was included in the models and the results supported our hypotheses, future studies are still warranted to further confirm the findings in a cohort with uncontrolled hypertension. We here focused on older adults with age >60 years, and previous studies have linked the cardiovascular risk in mid-aged population to the abnormalities of the brain (30, 31). It is thus of great significance to explore the relationship between the dynamics of BP and the functional outcomes in people at mid age. The multiscale dynamics of BP was still quantified within a relatively narrow range of time scales (i.e., 1–5). Thus, it is worthwhile to implement a longer BP recording time in future studies, allowing the observation of multiscale dynamics on a wider range of time scales. The WMLs were assessed by only the Fazekas scale, a commonly used scale in the clinics. It is warranted to implement other types of MRI techniques, such as diffusion-weighted MRI, together with advanced automated WML detection algorithm to quantify the WMLs more specifically (32), allowing the exploration of the relationship between BP complexity and subtle changes in cerebral regions. Another limitation is that the hypertension-induced target organ damage (TOD) (e.g., left ventricular hypertrophy, microalbuminuria) was not assessed in this pilot study. The TODs have been linked to the cerebrovascular damages (33, 34). It is thus worthwhile in future studies to specifically measure the TOD, which will help further understand how the multiscale dynamics of BP fluctuation is influenced in the pathology of hypertension, in which TODs may play an important role, especially in people with the most recent diagnosis of hypertension (35). Nevertheless, this study demonstrated that the BP complexity may be of great promise to help characterize the cardiovascular and cerebrovascular function in older adults and to provide novel insights into the age-related conditions related to the cardiovascular and cerebrovascular systems.

## Data Availability Statement

The raw data supporting the conclusions of this article will be made available by the authors, without undue reservation.

## Ethics Statement

The studies involving human participants were reviewed and approved by Institutional Review Board (IRB) of Shenzhen People's Hospital. The patients/participants provided their written informed consent to participate in this study.

## Author Contributions

XJ, YG, and JZ designed the study. XJ, XG, DP, HZ, WD, WF, NQ, and RC collected the data. XJ, YG, XG, YZ, HZ, and JZ analyzed the data and performed statistical analyses. XJ, YG, JZ, BM, and LL interpreted the results and drafted the manuscript. All authors contributed to and approved the final version.

## Conflict of Interest

The authors declare that the research was conducted in the absence of any commercial or financial relationships that could be construed as a potential conflict of interest.

## References

[1] Kim KW MacFall JR Payne ME. Classification of white matter lesions on magnetic resonance imaging in elderly persons. Bioll Psychiat. (2008) 64:273–80. 10.1016/j.biopsych.2008.03.024 18471801 PMC2593803

[2] Liao D Cooper L Cai J Toole J Bryan N Burke G. The prevalence and severity of white matter lesions, their relationship with age, ethnicity, gender, and cardiovascular disease risk factors: the ARIC Study. Neuroepidemiology. (1997) 16:149–62. 10.1159/000368814 9159770

[3] Prins ND van Dijk EJ den Heijer T Vermeer SE Koudstaal PJ Oudkerk M. Cerebral white matter lesions and the risk of dementia. Arch Neurol. (2004) 61:1531–4. 10.1001/archneur.61.10.1531 15477506

[4] de Groot JC de Leeuw FE Oudkerk M Hofman A Jolles J Breteler MM. Cerebral white matter lesions and depressive symptoms in elderly adults. Arch Gen Psychiat. (2000) 57:1071–6. 10.1001/archpsyc.57.11.1071 11074873

[5] Srikanth V Beare R Blizzard L Phan T Stapleton J Chen J. Cerebral white matter lesions, gait, and the risk of incident falls: a prospective population-based study. Stroke. (2009) 40:175–80. 10.1161/STROKEAHA.108.524355 18927448

[6] Levy BI Ambrosio G Pries AR Struijker-Boudier HA. Microcirculation in hypertension: a new target for treatment? Circulation. (2001) 104:735–40. 10.1161/hc3101.091158 11489784

[7] van Swieten JC Geyskes GG Derix MA Peeck BM Ramos LM Van Latum JC. Hypertension in the elderly is associated with white matter lesions and cognitive decline. Ann Neurol. (1991) 30:825–30. 10.1002/ana.410300612 1789694

[8] de Leeuw FE de Groot JC Oudkerk M Witteman JC Hofman A Van Gijn J. Hypertension and cerebral white matter lesions in a prospective cohort study. Brain. (2002) 125:765–72. 10.1093/brain/125.4.765 11912110

[9] Lipsitz LA. Age-related changes in the complexity of cardiovascular dynamics: a potential marker of vulnerability to disease. Chaos. (1995) 5:102–9. 10.1063/1.166091 12780162

[10] Dampney RA Coleman MJ Fontes MA Hirooka Y Horiuchi J Li YW. Central mechanisms underlying short-and long-term regulation of the cardiovascular system. Clin Exp Pharmacol Physiol. (2002) 29:261–68. 10.1046/j.1440-1681.2002.03640.x 11985533

[11] Costa M Goldberger AL Peng CK. Multiscale entropy analysis of complex physiologic time series. Phys Rev Lett. (2002) 89:068102. 10.1103/PhysRevLett.89.068102 12190613

[12] Zhou J Poole V Wooten T Lo OY Iloputaife I Manor B. Multi-scale dynamics of spontaneous brain activity is associated with walking speed in older adults. J Gerontol Series A. (2020) 75:1566–71. 10.1093/gerona/glz231 PMC7357585 31585008

[13] Yamashina A Tomiyama H Takeda K Tsuda H Arai T Hirose K. Validity, reproducibility, and clinical significance of noninvasive brachial-ankle pulse wave velocity measurement. Hypertens Res. (2002) 25:359–64. 10.1291/hypres.25.359 12135313

[14] Fazekas F Barkhof F Wahlund LO Pantoni L Erkinjuntti T Scheltens P. CT and MRI rating of white matter lesions. Cerebrovasc Dis. (2002) 13:31–6. 10.1159/000049147 11901240

[15] Guelen I Westerhof BE van der Sar GL van Montfrans GA Kiemeneij F Wesseling KH. Validation of brachial artery pressure reconstruction from finger arterial pressure. J Hypertens. (2008) 26:1321–7. 10.1097/HJH.0b013e3282fe1d28 18551006

[16] Costa M Goldberger AL Peng CK. Multiscale entropy analysis of biological signals. Phys Rev E. (2005) 71:021906. 10.1103/PhysRevE.71.021906 15783351

[17] Zhou J Lipsitz L Habtemariam D Manor B. Sub-sensory vibratory noise augments the physiologic complexity of postural control in older adults. J Neuroeng Rehabil. (2016) 13:44. 10.1186/s12984-016-0152-7 27142280 PMC4855814

[18] Williamson JD Pajewski NM Auchus AP Bryan RN Chelune G. Effect of intensive vs. standard blood pressure control on probable dementia: a randomized clinical trial. JAMA. (2019) 321:553–61. 30688979 10.1001/jama.2018.21442 PMC6439590

[19] Qiu C Winblad B Fratiglioni L. The age-dependent relation of blood pressure to cognitive function and dementia. Lancet Neurol. (2005) 4:487–99. 10.1016/S1474-4422(05)70141-1 16033691

[20] Bristow JD Honour AJ Pickering GW Sleight P Smyth HS. Diminished baroreflex sensitivity in high blood pressure. Circulation. (1969) 39:48–54. 10.1161/01.CIR.39.1.48 4302539

[21] Di Rienzo M Parati G Radaelli A Castiglioni P. Baroreflex contribution to blood pressure and heart rate oscillations: time scales, time-variant characteristics and nonlinearities. Philos T Roy Soc A. (2009) 367:1301–18. 10.1098/rsta.2008.0274 19324710 PMC2635500

[22] Manor B Costa MD Hu K Newton E Starobinets O Kang HG. Physiological complexity and system adaptability: evidence from postural control dynamics of older adults. J Appl Physiol. (2010) 109:1786–91. 10.1152/japplphysiol.00390.2010 20947715 PMC3006415

[23] Kaplan DT Furman MI Pincus SM Ryan SM Lipsitz LA Goldberger AL. Aging and the complexity of cardiovascular dynamics. Biophys J. (1991) 59:945–9. 10.1016/S0006-3495(91)82309-8 2065195 PMC1281262

[24] Schulz S Koschke M Bär KJ Voss A. The altered complexity of cardiovascular regulation in depressed patients. Physiol Meas. (2010) 31:303. 10.1088/0967-3334/31/3/003 20086275

[25] Henriques TS Costa MD Mathur P Mathur P Davis RB Mittleman MA. Complexity of preoperative blood pressure dynamics: possible utility in cardiac surgical risk assessment. J Clin Monitor Comp. (2019) 33:31–8. 10.1007/s10877-018-0133-4 29564751 PMC6150848

[26] Trunkvalterova Z Javorka M Tonhajzerova I Javorkova J Lazarova Z Javorka K. Reduced short-term complexity of heart rate and blood pressure dynamics in patients with diabetes mellitus type 1: multiscale entropy analysis. Physiol Meas. (2008) 29:817. 10.1088/0967-3334/29/7/010 18583725

[27] Longstreth WT Manolio TA Arnold A Burke GL Bryan N Jungreis CA. Clinical correlates of white matter findings on cranial magnetic resonance imaging of 3301 elderly people: the Cardiovascular Health Study. Stroke. (1996) 27:1274–82. 10.1161/01.STR.27.8.1274 8711786

[28] van Dijk EJ Breteler MM Schmidt R Berger K Nilsson LG Oudkerk M. The association between blood pressure, hypertension, and cerebral white matter lesions: cardiovascular determinants of dementia study. Hypertension. (2004) 44:625–30. 10.1161/01.HYP.0000145857.98904.20 15466662

[29] de Leeuw FE de Groot JC Oudkerk M Witteman JC Hofman A Van Gijn J. A follow-up study of blood pressure and cerebral white matter lesions. Ann Neurol. (1999) 46:827–33. 10.1002/1531-8249(199912)46:6<827::aid-ana4>3.3.co;2-8 10589534

[30] Knopman D Boland LL Mosley T Howard G Liao D Szklo M. Cardiovascular risk factors and cognitive decline in middle-aged adults. Neurology. (2001) 56:42–8. 10.1212/WNL.56.1.42 11148234

[31] Whitmer RA Sidney S Selby J Johnston SC Yaffe K. Midlife cardiovascular risk factors and risk of dementia in late life. Neurology. (2005) 64:277–81. 10.1212/01.WNL.0000149519.47454.F2 15668425

[32] Jones DK Knösche TR Turner R. White matter integrity, fiber count, and other fallacies: the do's and don'ts of diffusion MRI. Neuroimage. (2013) 73:239–54. 10.1016/j.neuroimage.2012.06.081 22846632

[33] Selvetella G Notte A Maffei A Calistri V Scamardella V Frati G. Left ventricular hypertrophy is associated with asymptomatic cerebral damage in hypertensive patients. Stroke. (2003) 34:1766–70. 10.1161/01.STR.0000078310.98444.1D 12805496

[34] Umemura T Kawamura T Sakakibara T Mashita S Hotta N Sobue G. Microalbuminuria is independently associated with deep or infratentorial brain microbleeds in hypertensive adults. Am J Hypertens. (2012) 25:430–6. 10.1038/ajh.2011.254 22237153

[35] Buono F Crispo S Pagano G Rengo G Petitto M Grieco F. Determinants of left ventricular hypertrophy in patients with recent diagnosis of essential hypertension. J Hypertens. (2014) 32:166–73. 10.1097/HJH.0b013e328365c87d 24126712

