# Supplementary figures and images for: Multiscale Dynamics of Blood Pressure Fluctuation Is Associated With White Matter Lesion Burden in Older Adults With and Without Hypertension: Observations From a Pilot Study
Source: Front Cardiovasc Med. 2021 Feb 26;8:636702. doi: 10.3389/fcvm.2021.636702 (PMC7952298; doi:10.3389/fcvm.2021.636702)

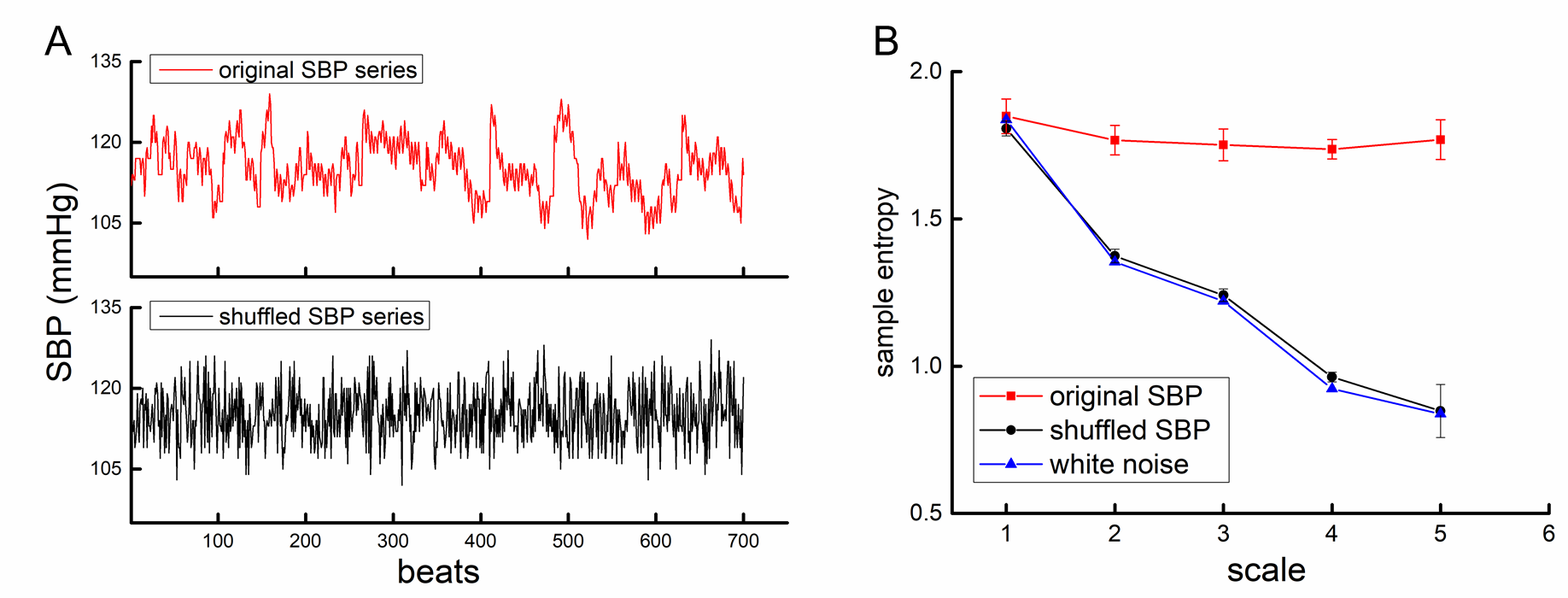

Supplement: Supplementary file 1 [file Image_1.TIF]
