# Supplementary material for: Multiscale Dynamics of Blood Pressure Fluctuation Is Associated With White Matter Lesion Burden in Older Adults With and Without Hypertension: Observations From a Pilot Study
Source: Front Cardiovasc Med. 2021 Feb 26;8:636702. doi: 10.3389/fcvm.2021.636702 (PMC7952298; doi:10.3389/fcvm.2021.636702)
Supplement: Supplementary file 2 [file Presentation_1.PDF]

Supplementary Materials

To further ensure the validity of complexity metric quantified by MSE, we performed the analysis on a 'surrogate' series of SBP created by shuffling the original SBP series (Figure S1). As expected, the MSE curve produced by surrogate data was similar to the MSE curve produced from random white noise, that is, the entropy was initially high at small scales, but decreased with the increase of scales. On the other hand, averaged MSE curves derived from the original SBP series were visibly different from those created from surrogate data. Thus, the estimated complexity reflected the intrinsic multi-scale dynamics of continuous beat-to-beat BP fluctuations rather than a random pattern.

Figure S1. The original SBP series (red) had greater complexity than the surrogate one (black). A. An example of the original SBP series (upper left panel) and its surrogate shuffled data (lower left panel). B shows the results of MSE from the original and shuffled BP series in comparison to white noise. The entropies of shuffled time series decrease as a function of increasing scales, which is similar to the white noise's MSE pattern (blue), indicating that the intrinsic physiologic mechanisms influencing the dynamics of BP were disrupted due to shuffling. As compared to the shuffled time series, the original SBP series had greater entropies than the shuffled or white noise data at larger scales, indicating that the original SBP series was more complex, with meaningful physiologic inputs, compared to the shuffled ones.
